# Health Implications of Inadequate Follow-Up for Women on Hormone Replacement Therapy in Primary Care: A Questionnaire-Based Cross-Sectional Study

**DOI:** 10.7759/cureus.96111

**Published:** 2025-11-04

**Authors:** Sara Elsaadany, Ashweta Josan

**Affiliations:** 1 Emergency Medicine/Family Medicine, North East London NHS Foundation Trust, London, GBR; 2 Family Medicine, North East London NHS Foundation Trust, London, GBR

**Keywords:** general practice, gynaecology and obstetrics, hormone replacement therapy (hrt), nice guidelines, postmenopause

## Abstract

Background

Hormone replacement therapy (HRT) is widely used to alleviate menopausal symptoms and improve quality of life. However, its long-term safety and efficacy depend on regular follow-up to monitor for adverse effects, reassess treatment goals, and ensure adherence to clinical guidelines. Evidence suggests a gap in the provision of appropriate follow-up care for women on HRT, particularly in primary care settings.

Objective

This questionnaire-based cross-sectional study aims to evaluate the extent of follow-up care provided to women on HRT and explore the health implications of inadequate monitoring, including symptom control, safety concerns, and patient outcomes, in a large primary care practice in East London. By identifying gaps in monitoring and the consequences of inadequate follow-up, this study seeks to develop strategies to improve follow-up for women on HRT, ensuring patient safety and optimal symptom management.

Methods

Patients who had been initiated on HRT between 2021 and 2024 were identified using electronic patient records (EPRs). A structured questionnaire was sent to assess symptom control, identify red-flag symptoms suggestive of malignancy, and gather insights into patients' concerns or questions regarding their treatment. Data was then collected on follow-up practices in an Excel spreadsheet and analysed.

Results

The review revealed that none of the patients initiated on HRT received follow-up care in accordance with the National Institute for Health and Care Excellence (NICE) guidelines and no annual reviews were conducted. Notably, 43% (N=84) of patients expressed uncertainty regarding the recommended duration of HRT use. Additionally, 25% (N=49) reported inadequate symptom management, while 1.7% (N=3) exhibited red-flag symptoms warranting further investigation. Furthermore, 2% (N=4) of patients were found to be using HRT incorrectly, highlighting the need for improved patient education and follow-up care.

Conclusion

These findings expose a significant gap in follow-up care for women on HRT, with serious implications for both patient safety and treatment effectiveness. In the absence of adequate monitoring, issues such as inappropriate HRT use can go undetected, potentially compromising patient safety. To address this gap, structured follow-up protocols and improved communication in primary care are needed. Ensuring that women receive the guidance and support they need will be essential in making HRT both safer and more effective.

## Introduction

Menopause is a natural physiological transition marking the end of menstruation secondary to a decline in oestrogen levels and is usually diagnosed after 12 months of amenorrhea. While the median age of onset is 51, a small proportion of women experience menopause before the age of 40 due to premature ovarian insufficiency. Menopause is associated with a range of symptoms, including vasomotor instability, such as hot flushes and night sweats, urogenital symptoms such as vaginal dryness and decreased libido, and psychological manifestations, such as anxiety and depression. The severity of symptoms can vary widely among women [1,2].

Overall, the impact of menopause has been shown to have a negative impact on the health-related quality of life (HRQoL) with the most prominent influence coming from physical health-related symptoms [3]. These symptoms extend to the perimenopausal stage, the years leading to menopause, and the postmenopausal stage, leading some women to experience symptoms for more than 10 years. Furthermore, the hormonal changes associated with menopause increase the risk of long-term health conditions, such as cardiovascular disease and osteoporosis, highlighting the importance of appropriate management. For many women, medical intervention is needed to effectively manage these symptoms and maintain quality of life [4,5].

Hormone replacement therapy (HRT) remains the cornerstone for managing menopausal symptoms. It offers protective benefits against osteoporosis, reducing the fracture risk by 26%, and cardiovascular diseases with it being most cardio-protective when taken before the age of 60 and for six years or more [4,5]. However, its use is not without risks, such as venous thromboembolism, cardiovascular events, and hormone-sensitive malignancies [6]. 

HRT can be administered in the form of oestrogen or progesterone or combined in different formulations and methods. Oestrogen-only therapy is used for women who have undergone a hysterectomy; otherwise, combined therapy is offered to the remaining population as progesterone is essential in preventing endometrial hyperplasia. Oestradiol tablets or synthetic conjugates taken orally are the most researched and convenient form. Transdermal patches also work very well and have the benefit of going past the first-pass hepatic metabolic stage. The risk of venous thromboembolism and stroke is not as significantly increased with transdermal routes compared to oral and should be recommended to those who are at risk. Lastly, oestrogen can also be given via vaginal delivery through an inserted ring or creams. This formulation manages vulvovaginal symptoms without increasing systemic oestrogen levels and can be given without progesterone [6].

To balance these risks and benefits, the National Institute for Health and Care Excellence (NICE) guidelines recommend regular follow-up, including annual reviews to assess efficacy, manage side effects, and reassess risks [7]. Despite these recommendations, evidence indicates significant gaps in the follow-up care, particularly in primary care settings.

This study was undertaken to systematically evaluate the extent and quality of follow-up care provided to women on HRT in a large primary care practice in East London, assess adherence to NICE guidelines, and determine the health implications of the lack of follow-up. Furthermore, the study seeks to identify and explore potential strategies to enhance patient follow-up, thereby ensuring the more effective management of menopausal symptoms and mitigating the associated risks of HRT.

## Materials and methods

Data collection

**Figure 1:**
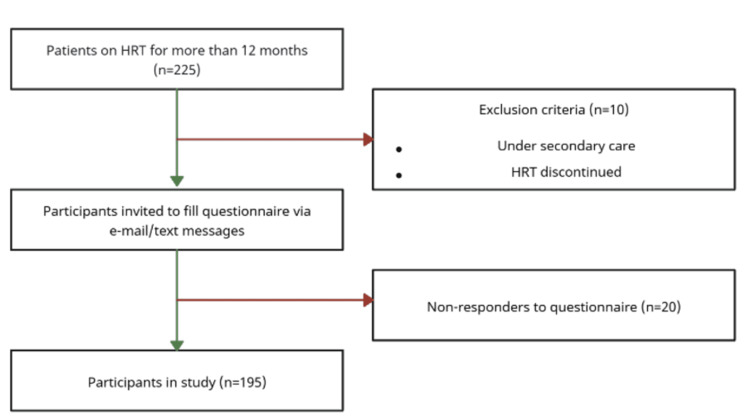
Flowchart illustrating the inclusion and exclusion criteria HRT: hormone replacement therapy

A questionnaire-based cross-sectional study was conducted in 2024 at the North East London Primary Care Centre in the United Kingdom. Informed consent was obtained from participants, and data were collected from women who had initiated HRT at least 12 months prior (N=225), as identified through EMIS, the electronic medical information system used in primary care. In this study, we did not apply exclusion criteria related to age or comorbidities, as the NICE guidelines for HRT follow-up in primary care are intended to encompass patients across all age groups, comorbidity profiles, and stages of menopause. Participants were identified through electronic patient records (EPRs) and invited to complete a structured survey via text and e-mail (N=215). Patients who had already stopped HRT or were under secondary care for the further management of their menopausal symptoms were excluded (N=10). Twenty patients did not respond to the survey making the total number of patients who took part 195 (N=195) (Figure 1).

The survey assessed several key areas, including symptom control to evaluate the persistence of menopausal symptoms, the presence of red-flag symptoms such as abnormal uterine bleeding, and the participants' understanding of HRT, particularly the recommended duration of therapy (Table 1).

**Table 1:** HRT questionnaire sent to patients HRT: hormone replacement therapy

HRT questionnaire
Demographics
Patient name
Patient age
Name of HRT
Duration of HRT
Symptom control
Are you suffering from vaginal dryness/discomfort?
Are you suffering from hot flushes?
Do you find that the HRT is helping with your symptoms?
Are you getting any side effects from HRT?
Risk and benefit
Is your breast screening up to date?
Is your cervical screening up to date?
What is your smoking status?
Any family history of breast cancer?
Any new unexpected vaginal bleeding/spotting?
Have you had a previous hysterectomy?
What is your BMI (height and weight)?
What is your most recent blood pressure measurement?
Other questions
Any new medication started?
Any change to health since the last review?
Are you on contraception?
What contraception are you using?
Any other questions related to HRT?

The questionnaire addresses key recommendations for follow-up as per the NICE guideline NG23 and the British Menopause Society (BMS). It includes elements advised in these guidelines, such as assessment of comorbidities (e.g., cardiovascular and thromboembolic risk), blood pressure monitoring, enquiry about abnormal bleeding, and confirmation of patient understanding of treatment risks and benefits. Its standardised structure and repeated use at each review support reproducibility and promote consistent, guideline-based monitoring of women receiving HRT in primary care. Additionally, the survey explored treatment adherence and HRT usage, along with any further questions the patients had [7,8]. 

Outcomes and statistical analysis

Data collected from the questionnaires were compiled in an Excel spreadsheet and analysed using Python (Python Software Foundation, Wilmington, DE, USA). Descriptive and inferential statistical analyses were performed to assess treatment adherence in relation to prescribed HRT, the adequacy of follow-up as defined by NICE guidelines, and associated clinical outcomes among women receiving HRT [7]. Descriptive statistics were used to summarise the baseline characteristics of the patient population, including demographics, prevalence of menopausal symptoms, presence of red-flag symptoms, and levels of treatment compliance. Categorical variables, such as the presence or absence of specific symptoms and compliance with treatment, were presented as frequencies and percentages. 

For inferential analysis, chi-squared tests were used to explore associations between categorical variables, such as the relationship between regular follow-up and symptom control or the presence of red-flag symptoms, with statistical significance set at p<0.05. The statistical analysis tested the null hypothesis that there is no significant association between the quality or adequacy of HRT follow-up in primary care and the risk of adverse outcomes being missed among women receiving HRT.

## Results

**Figure 2:**
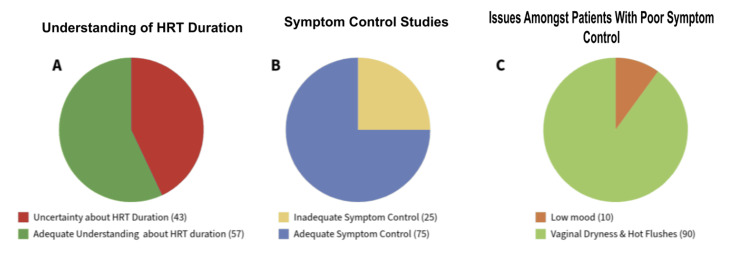
Patient-reported outcomes related to HRT The figure represents patient-reported outcomes related to HRT in percentages. Image A shows the percentage of patients and their understanding of HRT duration with 43% of patients reporting uncertainty about HRT duration and 57% of patients believing that they have an adequate understanding about HRT duration. Image B illustrates symptom control status on HRT with 25% reporting inadequate control and 75% reporting adequate control. Image C conveys the percentage of the two main issues identified with the patients reporting poor symptom control on HRT; 10% report low mood and 90% report vaginal dryness and hot flushes. HRT: hormone replacement therapy

Among the 195 patients evaluated, none (0%; N=0) received follow-up care consistent with NICE guidelines. The mean age of participants was 52 years (IQR 48-57). The duration of HRT use ranged from one to five years, with a mean duration of two years (IQR 1.5-3) (Table 2). Regarding patient-reported outcomes, 43% (N=84) were uncertain about the recommended duration of HRT, while 25% reported inadequate symptom control. A small proportion, 1.7% (N=3), exhibited red-flag symptoms warranting further evaluation, and 2% (N=4) were identified as using HRT incorrectly, highlighting significant gaps in patient education. Among those presenting with red-flag symptoms, spotting and vaginal bleeding were the most frequently reported. Of the patients reporting poor symptom control, 90% cited persistent vaginal dryness and hot flushes, whereas 10% (N=20) identified low mood as their predominant symptom (Figure 2).

**Table 2:** Demographics of the patients and HRT treatment details HRT: hormone replacement therapy; IQR: interquartile range

	Patients on HRT for more than one year (N=195)
Demographics	
Age, years (IQR)	52 (48-57)
HRT duration	
Length of treatment, years (IQR)	2 (1.8-3)
Sequential HRT, number of patients (%)	58 (29.74%)
Continuous HRT, number of patients (%)	137 (70.26%)
HRT formulation	
Oral oestrogen and progesterone, number of patients (%)	101 (52.79%)
Oral oestrogen alone, number of patients (%)	2 (1.02%)
Patches, number of patients (%)	67 (34.35%)
Gel, number of patients (%)	25 (12.80%)
Spray, number of patients (%)	0 (0%)

**Figure 3:**
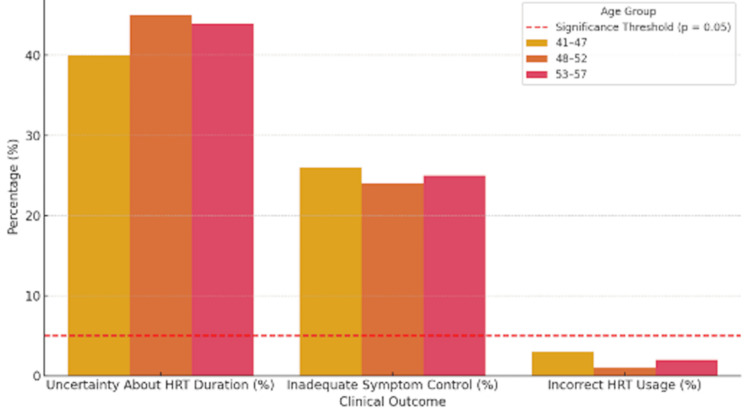
Bar graph representing the distribution of clinical outcomes HRT: hormone replacement therapy

To assess potential associations between demographic factors and clinical outcomes, chi-squared tests were conducted. Age was categorised into three groups (41-47 years, 48-52 years, and 53-57 years) to examine relationships with key variables, including uncertainty regarding HRT duration, inadequate symptom control, and incorrect HRT usage. No statistically significant association was observed between age group and uncertainty about HRT duration (χ²=1.89; p=0.38) or between age group and inadequate symptom control (χ²=2.45; p=0.29), suggesting that these challenges were evenly distributed across different age categories. A weak association was noted between age group and incorrect HRT use; however, this did not reach statistical significance (χ²=3.12; p=0.08), likely reflecting the small number of patients affected (Figure 3).

Given the limited sample size and the uniform absence of guideline-based follow-up, logistic regression analysis was considered to identify potential predictors of inadequate symptom control. Independent variables included age, duration of HRT use, and the absence of follow-up care. However, due to the small number of adverse events (1.7% with red-flag symptoms and 2% using HRT incorrectly) and the lack of variability in follow-up practices, there was insufficient power to detect meaningful associations. Despite these limitations, the descriptive and inferential analyses identify gaps in HRT management, particularly concerning patient education, and the implementation of structured follow-up protocols to improve clinical outcomes in primary care settings.

## Discussion

The results highlight a gap in the follow-up care of women initiated on HRT. This gap can be attributed to multiple factors, including limited healthcare resources and insufficient patient education regarding the importance of regular follow-up. The absence of appropriate follow-up is particularly concerning, as HRT, like any other medical therapy, carries potential risks and side effects that necessitate continuous monitoring and periodic risk reassessment. 

Moreover, the incorrect use of HRT observed in a subset of patients underscores a critical gap in patient education. Ensuring adherence to prescribed regimens is vital for achieving the desired therapeutic outcomes. Current guidelines recommend initiating HRT at low doses and titrating up until effective control of symptoms is achieved. Low doses improve tolerability and reduce cardiovascular and thromboembolic risks. This is particularly important for women with cardiovascular risk factors [9]. For women who begin HRT during the perimenopausal stage, a sequential regimen is recommended, in which progesterone is administered for up to 12 days each month to reduce withdrawal bleeding. Annual review is essential, as these women should be transitioned to a continuous regimen, where progesterone is given daily, after one to two years. Without proper follow-up, safe dose titration is not possible, potentially leaving women on unsuitable doses and regimens that compromise effectiveness and increase risk. Regular follow-up appointments, whether virtual or in person, serve as crucial touch points to reinforce correct usage, identify potential barriers to adherence, and provide targeted educational support [10].

Follow-up is also vital for reviewing treatment duration. Our study shows that 84 (43%) patients who participated in the questionnaire expressed uncertainty regarding the recommended duration of HRT. This finding suggests that some patients may be undergoing treatment for extended periods without appropriate review, potentially exposing them to unnecessary risks. The updated Women's Health Initiative (WHI) randomised trials published in 2017, with over 18 years of follow-up, showed HRT provided benefits that outweighed the risks of cardiovascular and cancer-related mortality, but only when given for a limited duration of 5.6-7.2 years. Prolonged use did not contribute to an additional mortality benefit, with the risks outweighing the benefits in long-term use with advancing age [11]. It highlights the need for clear communication between healthcare providers and patients about the intended duration of HRT, as well as the importance of scheduled follow-up visits to reassess the ongoing need for therapy.

Our findings of inconsistent follow-up and gaps in HRT management mirror concerns highlighted by Hamoda et al. and the All-Party Parliamentary Group (APPG) report regarding the "postcode lottery" in menopause care. Our study showed variability in the guidance and support regarding HRT duration. Despite increased awareness and reduced stigma surrounding HRT, these issues suggest that consistent, guideline-adherent guidance and follow-up have yet to be fully established in primary care [12].

Lastly, it is crucial to identify red-flag symptoms that could represent an underlying malignancy such as spotting or postmenopausal bleeding, as those would require further evaluation under the two-week wait to rule out an underlying endometrial malignancy. In our study, a small proportion of women reported experiencing such symptoms but had not disclosed them, highlighting the importance of proactive enquiry during follow-up consultations. Furthermore, patients should be educated on the importance of attending routine cervical and breast cancer screening, particularly given the potential increased risk of these malignancies with prolonged oestrogen use. These discussions should take place both at treatment initiation and during follow-up appointments to ensure early detection [10].

## Conclusions

In summary, this study highlighted gaps in the follow-up care provided to patients receiving HRT within primary care. None of the participants received reviews consistent with NICE recommendations, reflecting a broader issue of limited implementation of guideline-based follow-up in general practice. This lack of structured review may contribute to suboptimal symptom control, incorrect HRT use, and uncertainty among patients regarding treatment duration and monitoring requirements. 

Although HRT remains the most effective treatment for vasomotor and urogenital symptoms associated with menopause and provides protective benefits for bone and cardiovascular health when used appropriately, its safe and effective use requires periodic reassessment of risks and benefits. Regular follow-up enables clinicians to review symptom response, identify adverse effects or contraindications such as abnormal bleeding, and reinforce adherence to screening programmes for breast and cervical cancer as recommended by NICE.

The absence of significant associations between age, treatment duration, and key clinical outcomes suggests that challenges in HRT follow-up are systemic rather than limited to specific demographic groups. This highlights the importance of introducing consistent review mechanisms across all patient populations. Strategies such as incorporating automated recall systems within EPRs, adopting standardised templates for annual reviews, and enhancing patient education through accessible information resources could improve adherence to guidelines and promote shared decision-making.

While the findings should be interpreted in light of the study's limitations, including its single-centre design, small sample size, and reliance on self-reported data, they nonetheless provide useful insights into current gaps in primary care practice. Future research involving larger, multi-centre, and longitudinal studies would help to confirm these results and evaluate the effectiveness of interventions aimed at improving HRT follow-up.

Overall, improving the structure and consistency of HRT review in primary care has the potential to enhance the safety, efficacy, and patient experience of menopause management. A systematic approach, supported by clear communication and collaborative follow-up between patients and healthcare providers, may contribute to better long-term outcomes and closer alignment with national standards of care.
